# Supplementary material for: Ionic liquid gating induced self-intercalation of transition metal chalcogenides
Source: Nat Commun. 2023 Aug 16;14:4945. doi: 10.1038/s41467-023-40591-5 (PMC10432556; doi:10.1038/s41467-023-40591-5)
Supplement: Supplementary file 1 — Supplementary information [file 41467_2023_40591_MOESM1_ESM.pdf]

Supplementary materials

Ionic liquid gating induced self-intercalation of transition metal chalcogenides

Fei Wang[1,*], Yang Zhang[1,*], Zhijie Wang[2,*], Haoxiong Zhang[1], Xi Wu[2], Changhua Bao[1], Jia Li[2,†], Pu Yu[1,3,†] & Shuyun Zhou[1,3,†]

[1]*State Key Laboratory of Low Dimensional Quantum Physics and Department of Physics, Tsinghua University, Beijing 100084, People's Republic of China*

[2]*Shenzhen Geim Graphene Center and Institute of Materials Research, Tsinghua Shenzhen International Graduate School, Tsinghua University, Shenzhen, 518055, People's Republic of China*

[3]*Frontier Science Center for Quantum Information, Beijing 100084, People's Republic of China*

** These authors contributed equally to this work*

† Correspondence and request for materials should be sent to syzhou@mail.tsinghua.edu.cn, yupu@mail.tsinghua.edu.cn, li.jia@sz.tsinghua.edu.cn

Contents:

- Supplementary Note 1: Experimental setup and optimization of the self-intercalation conditions.
- Supplementary Note 2: Successful self-intercalation of $PdTe_2$ flake.
- Supplementary Note 3: More characterizations of the self-intercalation process of $PdTe_2$, and transport properties of $PdTe_2$ and PdTe.
- Supplementary Note 4: Characterization of the self-intercalation of $NiTe_2$.
- Supplementary Note 5: Characterization of exfoliated NiTe flake and transport measurements.

1 Experimental setup and optimization of the self-intercalation conditions.

The experimental setup is shown in Supplementary Fig. 1. Cyclic voltametric measurements of the intercalation process are shown in Supplementary Fig. 2. The current (or conductivity) starts to increase at the voltage of -1.9 V for $PdTe_2$ (-1.5 V for $NiTe_2$) suggesting that the single crystal starts dissolving into the ionic liquid. Upon further increasing the biased voltage to -3.2 V for $PdTe_2$ (-3.2 V for $NiTe_2$), the current increases rapidly, indicating that the intercalation reaction is in progress. We propose that the flow of ions leads to a current increase, while some ions will form compounds during this process, which will lead to a decrease in the concentration of conductive ions. When the voltage is beyond -3.6 V for $PdTe_2$ (-3.6 V for $NiTe_2$), most of the ions participate

in the formation of compounds, so there will be a stage of current decrease. If the voltage continues to increase to -3.8 V for $PdTe_2$ (-3.7 V for $NiTe_2$), the sample begins to decompose rapidly.

In order to optimize the experimental conditions, ex-situ Raman spectroscopy, X-ray diffraction (XRD) measurements were performed to monitor the extent of the intercalation at different time durations. From supplementary Fig. 3a, -3.2 eV is a good biased voltage, and Supplementary Figure 3b,c shows that after sufficient reaction time (72 hours), a complete transition of $PdTe_2$ into PdTe is achieved.

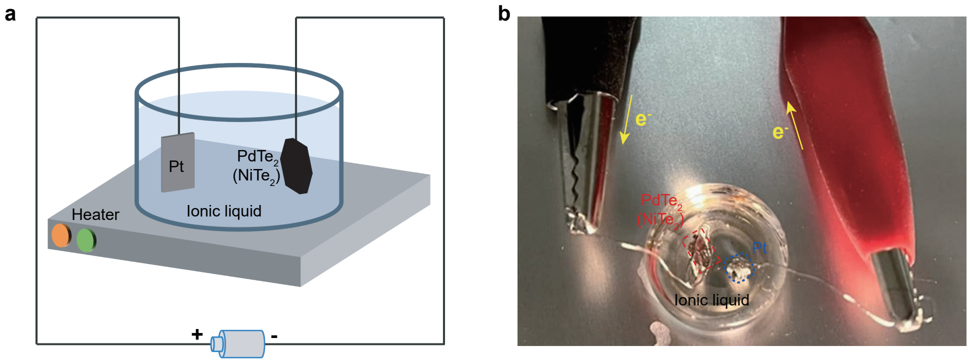

Supplementary Fig. 1 | Experimental setup for the self-intercalation. a, The schematic drawing of the experimental setup. **b**, Optical image of experimental setup employed for this study.

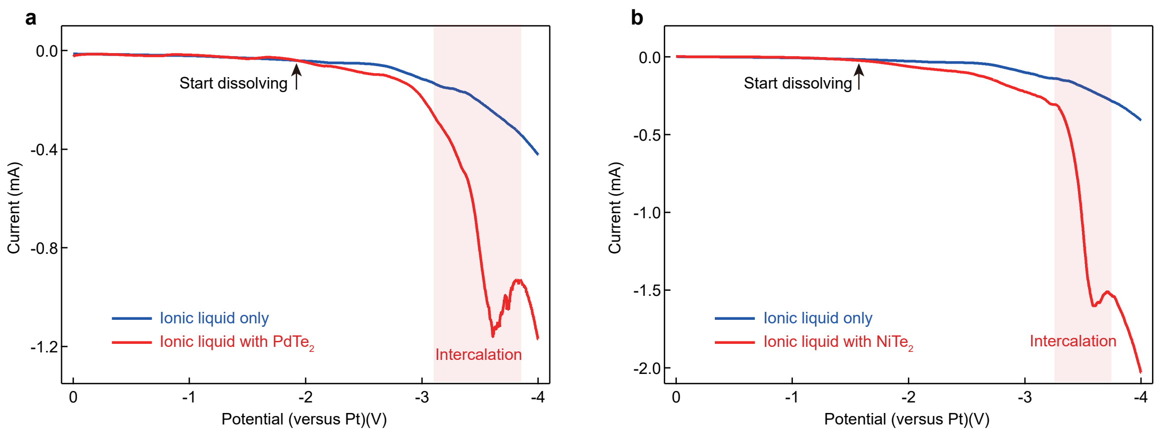

Supplementary Fig. 2| The characterization of intercalation process. a, b, Current-voltage (I-V) curves with ionic liquid only (blue curves) and ioic liquid with $PdTe_2$ sample (**a**) and $NiTe_2$ simple (**b**) at two different temperatures (red curves).

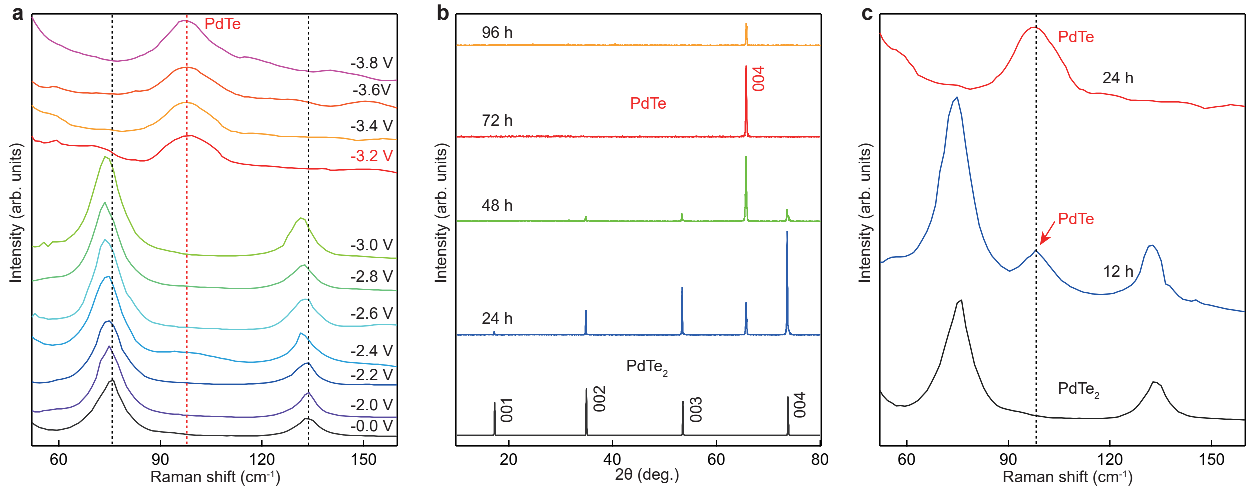

Supplementary Fig. 3 | Self-intercalation in $PdTe_2$ single crystal at different biased voltages. **a**, Raman spectra at different voltages. **b**, Detailed X-ray diffraction under different intercalation times, maintaining a biased voltage of -3.2 eV. **c**, Raman spectra after different intercalation times.

2 Successful self-intercalation of exfoliated $PdTe_2$ flake.

The self-intercalation of exfoliated flake can also be obtained using a similar setup (Fig. 2a). Figure 2b shows an optical image of the exfoliated $PdTe_2$ flake on Si/SiO_2 substrate, whose thickness is measured to be 170 nm by atomic force microscopy (AFM) measurements (Supplementary Fig. 4c, d). Supplementary Fig. 4e-h shows that after 8 hours (at 150 °C, -3.2 V) of ionic liquid gating, the $PdTe_2$ flake was successfully transformed into PdTe through intercalation, which is indicated by the characteristic PdTe Raman peak at 98 cm^{-1} (pointed by red arrow in Fig. 4e) and the uniform intensity map shown in Fig. 4g.

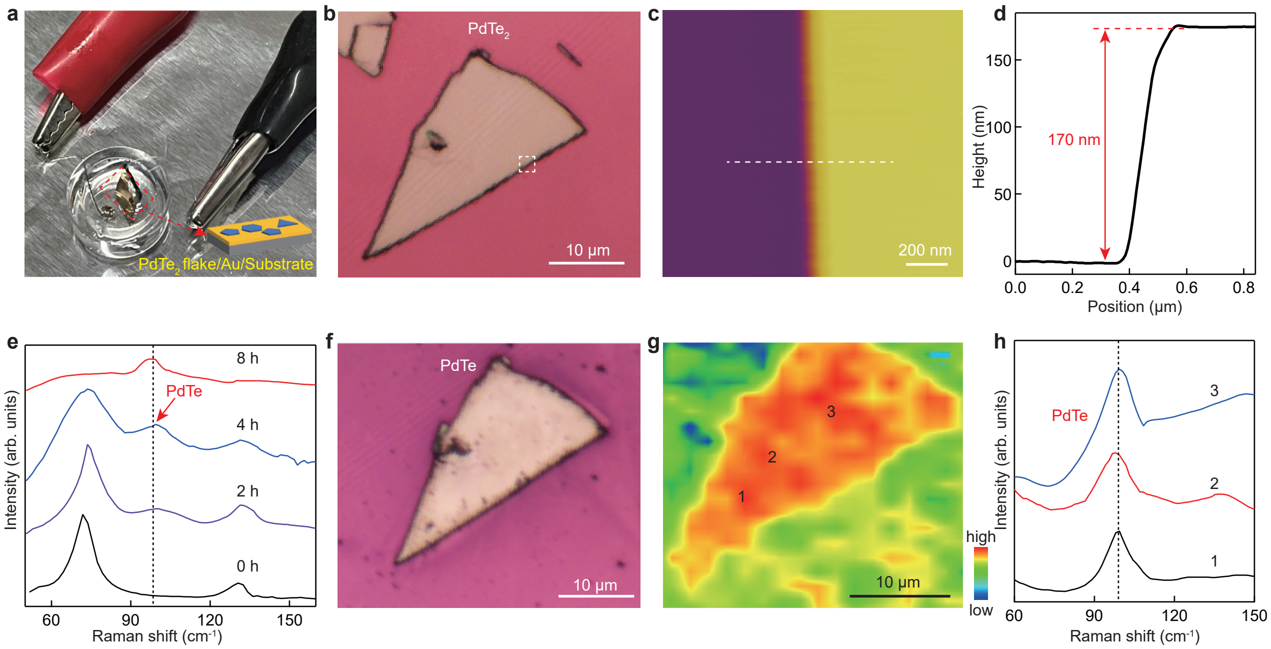

Supplementary Fig. 4 | The self-intercalation of $PdTe_2$ flake. a, Optical image of experimental setup for self-intercalation in $PdTe_2$ flake. **b, c**, The optical image (**b**) and AFM topography (**c**) of exfoliated $PdTe_2$ flake. **d**, The height of $PdTe_2$ flake extracted from AFM topography as indicated by the white line in (**c**). **e**, Raman spectra of $PdTe_2$ flake after different intercalation times. **f**, The optical image of flake after self-intercalation. **g**, Raman intensity mapping by integrating the characteristic mode 98 cm^{-1} of PdTe. **h**, Raman spectra acquired from location (1-3) at (**g**).

3 More characterizations of the self-intercalation process of $PdTe_2$, and transport properties of $PdTe_2$ and PdTe.

Supplementary Fig. 5 shows HAADF-STEM images and energy dispersive spectroscopy (EDS) measurements, which are used for extracting the lattice constants and analyzing the chemical compositions. Supplementary Fig. 6 shows a comparison of x-ray photoemission spectroscopy (XPS) measurements before and after self-intercalation of $PdTe_2$. A shift of the Te *3d* peak is clearly observed after intercalation.

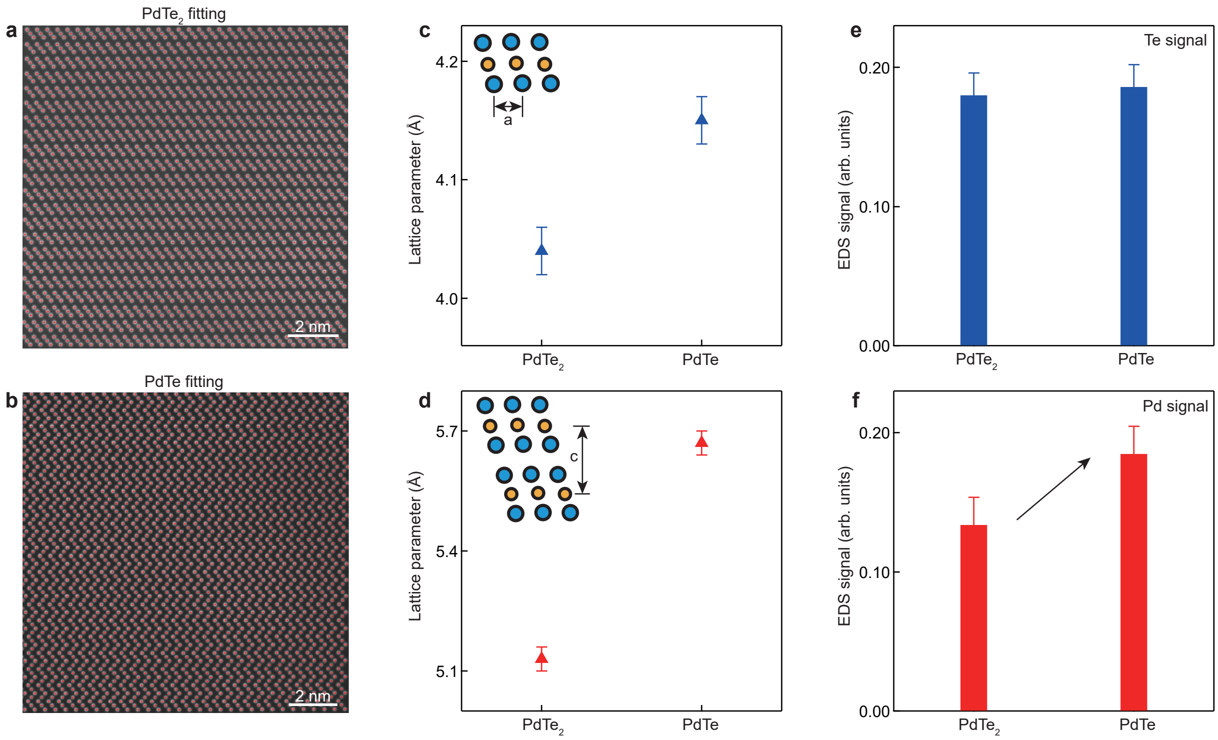

Supplementary Fig. 5| Evidence for self-intercalation of $PdTe_2$. **a, b**, Fitted atomic positions of $PdTe_2$ (a) and PdTe (b). **c, d**, The lattice constant of the a-axis and c-axis before and after self-intercalation. The inset shows the a-axis and c-axis schematic. The error bars of the lattice parameter are extracted from the fitting results. **e, f**, EDS signals of Te and Pd in $PdTe_2$ and PdTe respectively.The error bars are calculated from standard deviation of multiple sets of measurement data.

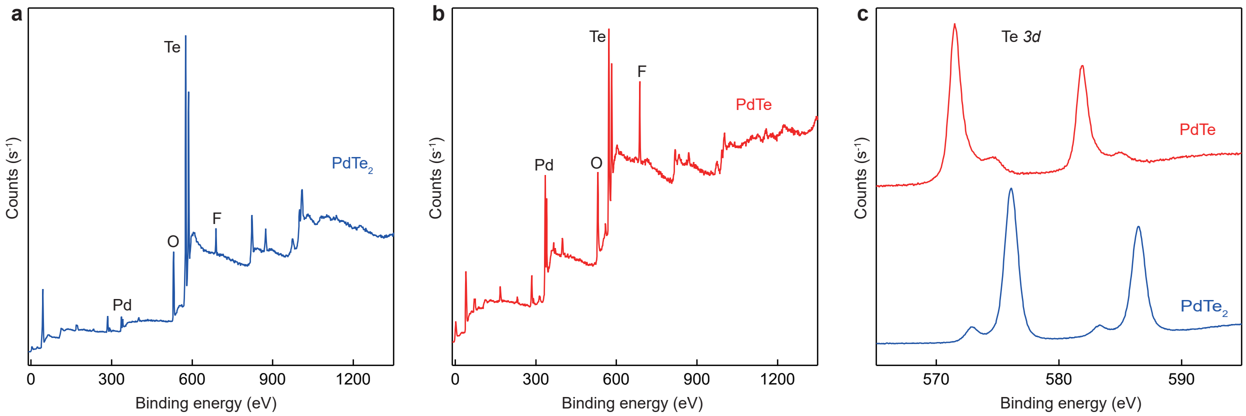

Supplementary Fig. 6 | XPS spectra of before and after self-intercalation. **a, b**, XPS spectra of the $PdTe_2$ (**a**) and PdTe (**b**). No elements other than Te, Pd, O and F were detected. **c**, Core level of Te *3d* in $PdTe_2$ and PdTe.

Supplementary Fig. 7 shows a comparison of transport measurements before and after self-intercalation of $PdTe_2$. Superconductivity is clearly observed in PdTe after the intercalation. Supplementary Fig. 8 shows transport measurements of PdTe samples obtained by using other ionic liquids as solvents, which shows that similar superconductivity can also be induced by self-intercalation of $PdTe_2$ using other ionic liquids.

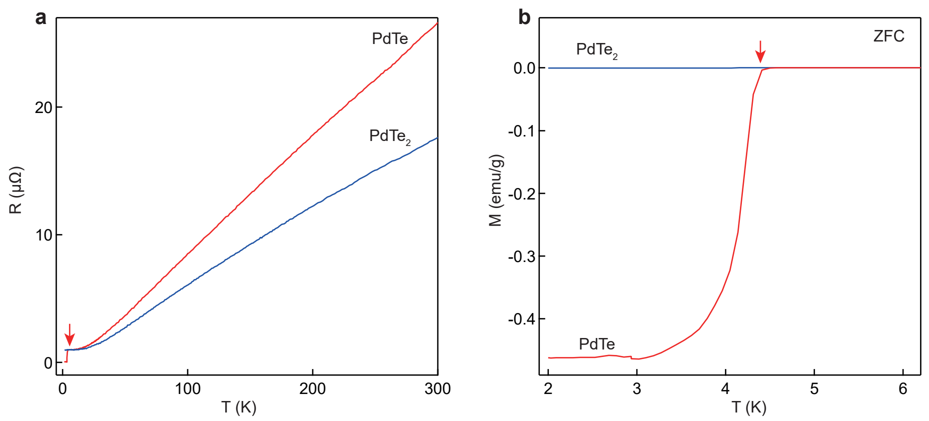

Supplementary Fig. 7 | Transport and magnetization measurements before and after self-intercalation. **a**, Temperature dependent resistance measurements on $PdTe_2$ and PdTe. **b**, Dc-magnetization recorded in ZFC for $PdTe_2$ and PdTe with 50 Oe applied magnetic field.

4 Characterization of the self-intercalation of $NiTe_2$.

Supplementary Fig. 9 shows the XRD and Raman measurements of the self-intercalation of $NiTe_2$ under different experimental conditions. The optimized voltage is - 3.4 V at 170 °C. The STEM measurements of a $NiTe/NiTe_2$ after self-intercalation are shown in Fig. 10.

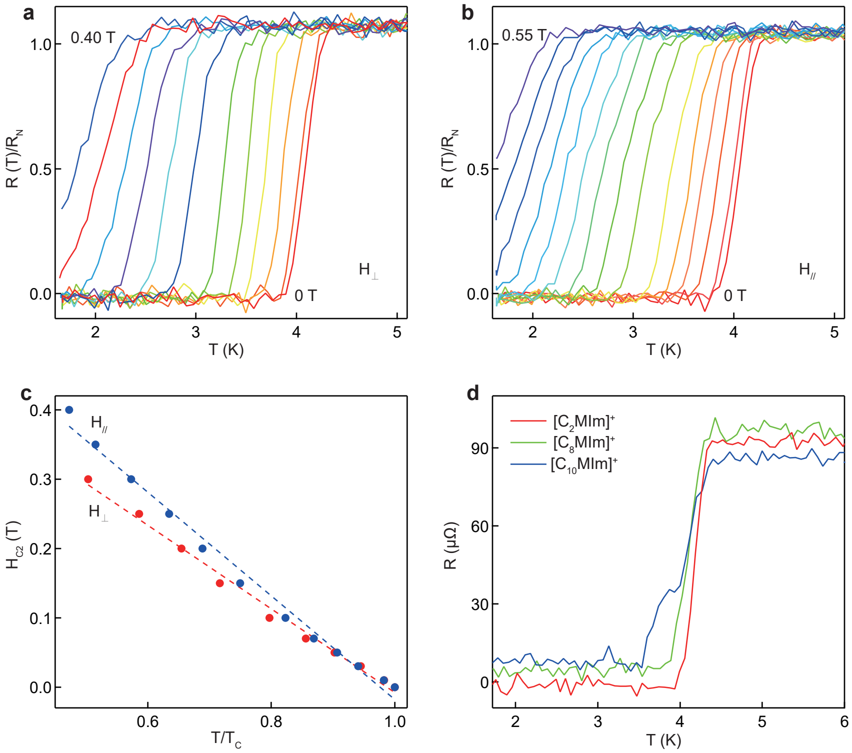

Supplementary Fig. 8 | The transport measurements of self-intercalated samples using other ionic liquids as solvents. a, b, Resistance of $[C_8MIm]^+$ gated sample under different out-of-plane magnetic fields and in-plane magnetic fields. The out-of-plane magnetic fields are 0, 0.01, 0.03, 0.05, 0.07, 0.1, 0.15, 0.2, 0.25, 0.3, 0.35, 0.4 T from red to purple curve. The in-plane magnetic fields are 0, 0.01, 0.03, 0.05, 0.07, 0.1, 0.15, 0.2, 0.25, 0.3, 0.35, 0.4, 0.45, 0.5, 0.55 T from red to purple curve. **c**, Extracted upper critical magnetic fields H_{C2} for $H_\perp$ and $H_{//}$ as a function of temperature. The dashed curves are fitted by Ginzburg-Landau equation. **d**, The superconductivity of self-intercalated sample after reaction in three different ionic liquids.

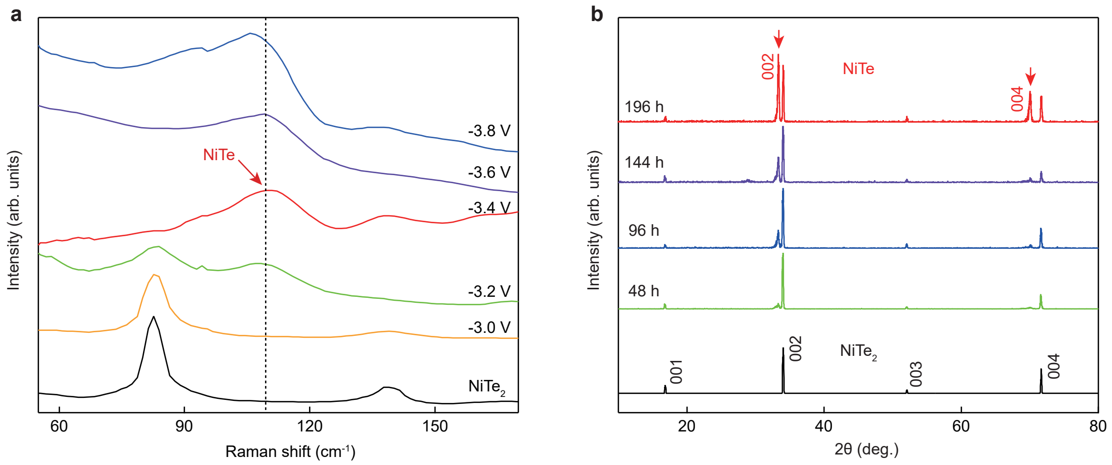

Supplementary Fig. 9 | Evidence for self-intercalation in $NiTe_2$ single crystal. a, Raman spectra at different voltages. **b**, Detailed X-ray diffraction under different intercalation times with biased voltage of -3.4 V.

5 Characterization of exfoliated NiTe flake.

Single phase NiTe flake can be exfoliated from the NiTe/$NiTe_2$ hetero-junction. To further confirm that the exfoliated flake is NiTe, we performed Raman mapping measurements on the back side of flake. Since the self-intercalation process occurs from the top surface, the Raman study on the back side of the exfoliated flake can directly confirm whether the exfoliated flake is NiTe. Supplementary Fig. 11a-c shows the Raman mapping on the back side of an exfoliated flake, in which the characteristic Raman peak at 109 cm^{-1} (Fig. 11c) and the uniform Raman intensity map (Fig. 11b) clearly confirm that the exfoliated flake is indeed NiTe. Supplementary Fig. 11d shows the exfoliated NiTe device for transport measurements, and the resistivity and magnetoresistance (MR) measurements are shown in Fig. 11e,f.

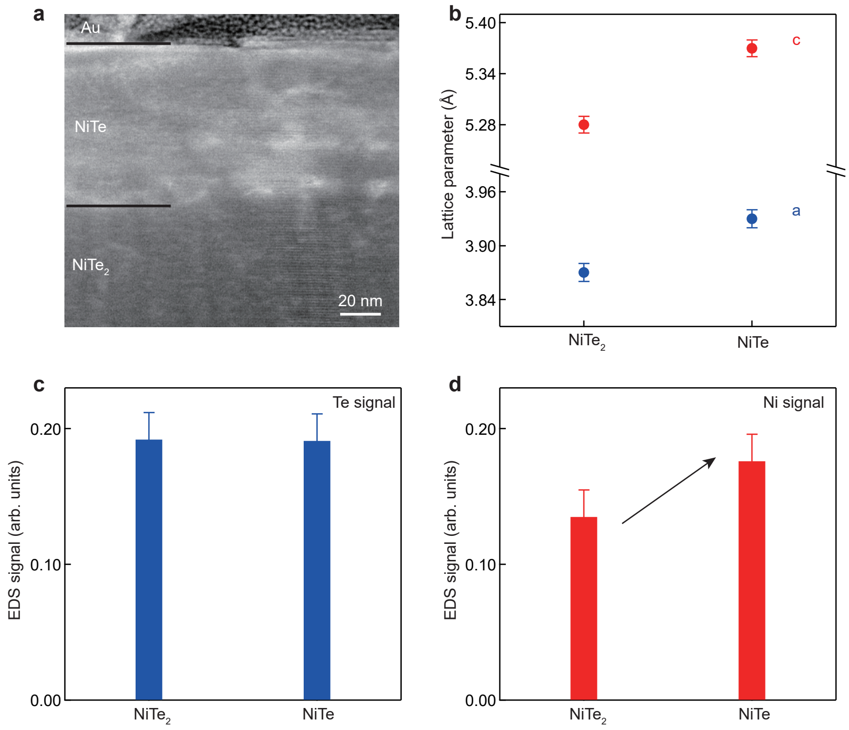

Supplementary Fig. 10 | STEM measurement for self-intercalation in $NiTe_2$ single crystal. **a**, Low-magnified HAADF-STEM image showing the heterostructure of $NiTe_2$ and self-intercalated NiTe. **b**, Measured lattice constants along the a-axis and c-axis. The inset shows the a-axis and c-axis schematic. The error bars of the lattice parameter are extracted from the fitting results. **c, d**, EDS signals of Pd and Te in $NiTe_2$ and NiTe respectively. The error bars are calculated from standard deviation of multiple sets of measurement data.

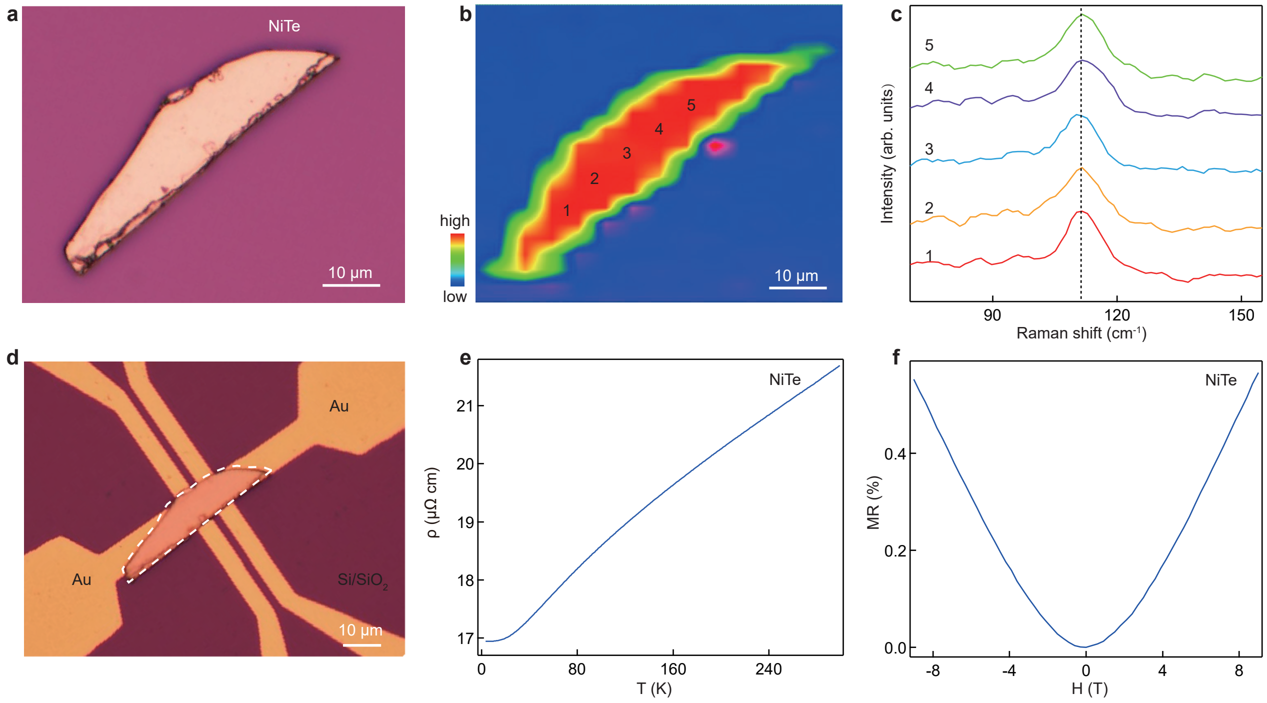

Supplementary Fig. 11 | Characterization of NiTe flake exfoliated from NiTe/$NiTe_2$ heterojunction and transport measurements. **a**, The optical photographs of exfoliated NiTe single crystal. **b**, Raman mapping by integrating the NiTe at 109 cm^{-1} mode. **c**, Raman spectra from location (1-5) at (**b**). **d**, Optical photographs of exfoliated NiTe after depositing on the pre-patterned Ti/Au (10 nm/50 nm) Hall bar on Si/SiO_2 substrate. **e, f**, Electrical and magnetoresistance measurements of exfoliated NiTe.
